# Feeding during neonatal therapeutic hypothermia, assessed using routinely collected National Neonatal Research Database data: a retrospective, UK population-based cohort study

**DOI:** 10.1016/S2352-4642(21)00026-2

**Published:** 2021-06

**Authors:** Chris Gale, Nicholas T Longford, Dusha Jeyakumaran, Kayleigh Ougham, Cheryl Battersby, Shalini Ojha, Jon Dorling

**Affiliations:** a Neonatal Medicine, School of Public Health, Faculty of Medicine, Imperial College London, Chelsea and Westminster Hospital campus, London, UK; b Neonatal Data Analysis Unit, School of Public Health, Faculty of Medicine, Imperial College London, Chelsea and Westminster Hospital campus, London, UK; c Division of Graduate Entry Medicine, School of Medicine, University of Nottingham, Nottingham, UK; d Division of Neonatal-Perinatal Medicine, Faculty of Medicine, Dalhousie University, IWK Health Centre, Halifax, NS, Canada

## Abstract

**Background:**

Therapeutic hypothermia is standard of care in high-income countries for babies born with signs of hypoxic ischaemic encephalopathy, but optimal feeding during treatment is uncertain and practice is variable. This study aimed to assess the association between feeding during therapeutic hypothermia and clinically important outcomes.

**Methods:**

We did a population-level retrospective cohort study using the UK National Neonatal Research Database. We included all babies admitted to National Health Service neonatal units in England, Scotland, and Wales between Jan 1, 2010, and Dec 31, 2017, who received therapeutic hypothermia for 72 h or died during this period. For analysis, we created matched groups using propensity scores and compared outcomes in babies who were fed versus unfed enterally during therapeutic hypothermia. The primary outcome was severe necrotising enterocolitis, either confirmed at surgery or causing death. Secondary outcomes include pragmatically defined necrotising enterocolitis (a recorded diagnosis of necrotising enterocolitis in babies who received at least 5 consecutive days of antibiotics while also nil by mouth during their neonatal unit stay), late-onset infection (pragmatically defined as 5 consecutive days of antibiotic treatment commencing after day 3), survival to discharge, measures of breastmilk feeding, and length of stay in neonatal unit.

**Findings:**

6030 babies received therapeutic hypothermia, of whom 1873 (31·1%) were fed during treatment. Seven (0·1%) babies were diagnosed with severe necrotising enterocolitis and the number was too small for further analyses. We selected 3236 (53·7%) babies for the matched feeding analysis (1618 pairs), achieving a good balance for all recorded background variables. Pragmatically defined necrotising enterocolitis was rare in both groups (incidence 0·5%, 95% CI 0·2–0·9] in the fed group *vs* 1·1% [0·7–1·4] in the unfed group). The enterally fed group had fewer pragmatically defined late-onset infections (difference −11·6% [95% CI −14·0 to −9·3]; p<0·0001), higher survival to discharge (5·2% [3·9–6·6]; p<0·0001), higher proportion of breastfeeding at discharge (8·0% [5·1–10·8]; p<0·0001), and shorter neonatal unit stays (−2·2 [–3·0 to −1·2] days; p<0·0001) compared with the unfed group.

**Interpretation:**

Necrotising enterocolitis is rare in babies receiving therapeutic hypothermia. Enteral feeding during hypothermia is safe and associated with beneficial outcomes compared with not feeding, although residual confounding could not be completely ruled out. Our findings support starting milk feeds during therapeutic hypothermia.

**Funding:**

UK National Institute for Health Research Health Technology Assessment programme 16/79/13.

## Introduction

Therapeutic hypothermia is standard of care in high-income countries for babies born at or after 36 weeks of gestational age with signs of hypoxic ischaemic encephalopathy.^1^ Optimal nutritional support for babies receiving therapeutic hypothermia is uncertain; previously published trials either did not specify nutritional management^2^ or stipulated that milk feeds should be withheld.^3^ In the absence of high-quality evidence, provision of enteral nutrition to infants during hypothermia is variable;^4^ withholding enteral feeds during therapeutic hypothermia is almost universal in some settings,^5^ whereas in other settings milk feeding is routine.^6^

Milk feeds are withheld during therapeutic hypothermia partly to reduce the risk of necrotising enterocolitis. The incidence of necrotising enterocolitis in term and near term infants with neonatal encephalopathy is poorly reported but is thought to be low.^2^, ^7^ Furthermore, some data suggest beneficial effects of enteral feeding after perinatal ischaemia.^8^

Given the context of variable clinical practice and a paucity of high-quality evidence, we aimed to identify optimal enteral feeding strategies for babies receiving therapeutic hypothermia. Because key outcomes, such as necrotising enterocolitis, are rare in this population of term and near-term babies, a randomised controlled trial powered to address such outcomes is not feasible. We therefore did an observational study using routinely recorded clinical data, and applied propensity-score matching to form groups for comparison that had nearly identical distributions of background variables.^9^, ^10^ We aimed to assess the association between enteral feeding during therapeutic hypothermia and the incidence of necrotising enterocolitis and other clinically important outcomes.

**Research in context**

**Evidence before this study**

We developed this study in 2017 and on July 7, 2017, searched PubMed using the search terms “therapeutic hypothermia” AND (“enteral” OR “nutrition”) without publication date or language restrictions, for articles describing feeding practices during neonatal therapeutic hypothermia. In the 11 trials included in the Cochrane review of hypothermia for hypoxic ischaemic encephalopathy, feeds were either withheld during therapeutic hypothermia, or feeding practice was either not stipulated or not reported. We found no published randomised controlled trials directly addressing this topic. One retrospective cohort study of 85 infants receiving therapeutic hypothermia compared practice in the UK where a majority of babies were not fed, and practice in Sweden where the majority of babies received milk feeds, and found no differences in clinical outcomes between countries and found no cases of necrotising enterocolitis. We repeated the PubMed search on Nov 12, 2020, using the same search terms. One retrospective, matched, case control study of 34 infants receiving therapeutic hypothermia in the USA compared inflammatory markers in babies that received minimal enteral nutrition versus babies who had feeds withheld, and found lower concentrations of inflammatory cytokines and fewer days of parenteral nutrition and days of hospital stay in the enteral feeding group. Surveys of practice in the UK, the USA, and Europe show that feeding practice during therapeutic hypothermia is variable.

**Added value of this study**

To our knowledge, our study analysed the largest cohort of babies who have received therapeutic hypothermia. Using national, population-level observational data, we produced and compared incidence data for necrotising enterocolitis, late-onset culture-positive bloodstream infection, and other outcomes between babies fed enterally versus babies from whom feeds were withheld during therapeutic hypothermia. We found that necrotising enterocolitis was rare, and enteral feeding during hypothermia treatment was safe and might be beneficial.

**Implications of all the available evidence**

These results support starting milk feeds in babies during therapeutic hypothermia, using maternal milk if available.

## Methods

### Study design and data source

We did a population-level, retrospective cohort study using routinely recorded neonatal clinical data held in the UK National Neonatal Research Database (NNRD). The NNRD^11^ holds data from all infants (approximately 90 000 annually) admitted to National Health Service (NHS) neonatal units in England, Scotland, and Wales. In the UK, therapeutic hypothermia is not provided outside of NHS neonatal units. Data held by the NNRD are extracted from neonatal electronic health records that have been completed by health professionals during routine clinical care. The neonatal dataset^12^ (a defined extract of approximately 450 data items) is transmitted quarterly to the Neonatal Data Analysis Unit, in which patient episodes across different hospitals are linked and the data are cleaned (ie, queries about discrepancies and implausible data configurations are fed back to health professionals and rectified). The completeness and accuracy of data held in the NNRD have been validated against case-record forms completed as part of a multicentre randomised controlled trial.^11^ NNRD data items include demographic and admission items (birthweight), daily items (feeding information), discharge items (weight at discharge), and ad-hoc items entered if and when they occur (eg, ultrasound scan findings).

The study was designed by a multiprofessional investigator group, which included a parent of a baby who received therapeutic hypothermia and a parent representative. Study outcomes were chosen to reflect those prioritised as important by parents, patients, and professionals,^13^ and were informed by parents and parent representatives. Research ethics approval for the study was obtained from East Midlands—Leicester Central Research Ethics Committee (17/EM/0307) and approval for inclusion of their data in this study was obtained from all English, Scottish, and Welsh neonatal units.

The study was prospectively registered (ISRCTN registry ISRCTN47404296; ClinicalTrials.gov NCT03278847) and the protocol has been published previously.^14^

### Participants

We extracted NNRD data for infants born between Jan 1, 2008, and Dec 31, 2017, who were admitted to NHS neonatal units in England, Scotland, and Wales that contributed data to the NNRD. Neonatal unit data from Jan 1, 2008 onwards, is held by the NNRD; from Jan 1, 2012 onwards, all NHS neonatal units in England and Wales have contributed data. Data were extracted for the duration of an infant's neonatal unit stay.

Infants were eligible for inclusion in the study if they had a recorded gestational age of 36 weeks or older at birth and were recorded either as having received therapeutic hypothermia for 3 days or as having died during that period after receipt of therapeutic hypothermia. UK national guidance^15^ during the study period recommended giving therapeutic hypothermia to babies with moderate to severe hypoxic ischaemic encephalopathy, as diagnosed in trials such as TOBY.^7^

### Procedures

We applied propensity-score methods to form matched subgroups to compare outcomes of infants with similar background characteristics but who had different enteral feeding strategies: babies that were enterally fed versus not enterally fed during therapeutic hypothermia. Being fed was defined as receiving milk feeds of any type, by any enteral route of administration, and in any quantity, for at least 1 day during the period of therapeutic hypothermia. Further details of codes used to define analysis groups are available in the appendix (p 2).

### Outcomes

The primary outcome was severe necrotising enterocolitis (using the UK Neonatal Collaborative definition^16^) confirmed at surgery or causing death, validated with the neonatal units. Secondary outcomes included pragmatically defined necrotising enterocolitis (defined as a recorded diagnosis of necrotising enterocolitis in babies who received at least 5 days consecutively of antibiotics while also nil by mouth during their neonatal unit stay); culture positive, late-onset bloodstream infection (defined using the UK National Neonatal Audit Programme case definition as a pure growth of a pathogen from blood^17^); suspected late-onset infection (defined as 5 days consecutively of antibiotic treatment commencing after day 3); survival to discharge from neonatal unit; length of neonatal unit stay; hypoglycaemia; breastfeeding at discharge; onset of breastfeeding (in babies who breastfed); age in days at receipt of first maternal milk (in babies that received maternal milk); days with a central line; duration of parenteral nutrition in days; and weight-for-age SD score at neonatal discharge. Further details of outcomes are available in the appendix (pp 2–5).

### Statistical analysis

The a-priori estimated sample size was 7200 babies, which was estimated to detect (two-sided 5% significance, power 90%) a difference of 0·7% in necrotising enterocolitis with 2000 matched pairs, assuming a negligible rate of necrotising enterocolitis in the not fed group.

To address potential confounders (eg, infants with multisystem disease, who might be more likely to have feeds withheld and also to have poorer outcomes than babies without multisystem disease), we used propensity score matching to form subgroups of infants with similar background characteristics (including clinical condition and treatment) when therapeutic hypothermia was started. The variables in the propensity model included demographic items (eg, gestational age, weight-for-age SD score, sex, birth multiplicity; maternal factors (eg, age, duration of rupture of membranes, fever, suspected chorioamnionitis, smoking status, ethnicity, deprivation score, hypothyroidism, diabetes, mode of delivery of infant, parity); factors at birth (eg, Apgar scores at 1 min and 5 min, chest compressions administered during resuscitation, emergency resuscitation drugs given, intubated at resuscitation, umbilical cord base excess, time to first spontaneous breath); condition on admission before therapeutic hypothermia (eg, mean blood pressure, glucose, heart rate, oxygen saturation, temperature); early-onset infection (eg, positive blood or cerebrospinal fluid culture with a recognised pathogen recorded in the first 3 days); treatment on day 1 (eg, infusion of inotropes, mechanical ventilation, and treatment with inhaled nitric oxide); and organisational factors (eg, postnatal transfer within 24 h, neonatal network of birth); further details can be found in the appendix (pp 2–5).

Analyses used the potential outcomes framework and propensity score method. We did 1:1 matching of babies that received no enteral feeds to babies who were fed enterally during therapeutic hypothermia. For each infant, the propensity of the nutritional exposure (enteral feeds or no enteral feeds) was estimated by logistical regression that included all background variables as covariates. Matched pairs were formed within these groups with one infant from either exposure group. Pairs were first matched by birth year (four 2 year bands) and arterial umbilical cord pH (three bands: <6·9, 6·9–7·0, and >7·0), making 12 groups in total. Matched pairs were then formed within propensity-score deciles defined separately for each background group. Because this matching procedure involves some randomness, it was replicated 25 times to produce 25 matched cohorts. Every subsequent analysis was done separately for each matched cohort and the replicated results were averaged to reduce the effect of the uncertainty involved in matching. A detailed description of the matching process is provided in the appendix (pp 9–11). The success of the matching process is shown by standardised differences of the background variables across each group before and after matching (see appendix pp 12–13).^18^ Outcomes in the resulting two matched subgroups were then compared by methods identical to those for a randomised trial, with absolute and relative risks of serious adverse outcomes derived. The two-sample t test was used for all comparisons of matched groups. For the odds ratio, the SE was estimated as described by Morris and Gardner.^19^ The SE of the estimate of the treatment effect was obtained by combining the within-replication SE and between-replication SE.^20^ All p values are two-sided.

We imputed data for babies who had records with missing data about receipt of therapeutic hypothermia on the second day, but who were recorded as having received therapeutic hypothermia on both the preceding and following days and did not die during cooling. No other data were imputed.

Two preplanned sensitivity analyses were done. First, analysis was restricted to babies born between Jan 1, 2012 and Dec 31, 2017, in England and Wales, during which period all NHS neonatal units in these two countries contributed data to the NNRD. This analysis was done to determine whether using the less complete NNRD data submitted before Jan 1, 2012 introduces bias. Second, analysis was restricted to infants for whom all enteral or parenteral feeding data have been actively recorded, by excluding infants with missing nutrition data during the first 4 days.

A third, post-hoc, sensitivity analysis was done (following the advice of the clinical investigator group and with the agreement of study steering committee) to examine the effect of nutritional practice on the first day as an additional background matching variable. In a final sensitivity analysis, receipt of parenteral nutrition on the first day after birth was added as a background variable to the propensity model. We accounted for multiple testing by applying the Bonferroni correction.

Before comparative analysis of matched groups, it became clear that the proportion of missing data was high in 2008 and 2009. The protocol was therefore altered and analysis was restricted to babies born between Jan 1, 2010 and Dec 31, 2017, for all analyses. The study was overseen by an independent study steering committee, which approved all deviations from protocol. Analyses were done using SAS (version 9.3) and R (version 3.3.1).

### Role of the funding source

This study was funded by the UK National Institute for Health Research (NIHR) Health Technology Assessment programme (16/79/13). The funder had no role in designing the study, the collection, analysis, or interpretation of the data, or in the writing of this Article.

## Results

Between Jan 1, 2010, and Dec 31, 2017, 703 907 babies were admitted to NHS neonatal units in England, Scotland, or Wales; 6030 were at least 36 weeks of gestational age and treated with therapeutic hypothermia for 3 days or died during treatment. 1873 (31·1%) of 6030 received enteral feeds during therapeutic hypothermia; this proportion changed only slightly between 2010 and 2017.

In the total study cohort of babies, and before matching, seven (0·1%) of 6030 babies that received therapeutic hypothermia were diagnosed with severe necrotising enterocolitis, and 68 (1·1%) of 6030 were classified as having necrotising enterocolitis using the pragmatic definition. 30 (0·5%) of 6030 babies had a pure growth of a recognised pathogen in a blood culture after day 3. Pragmatically defined late-onset infection was more common than pure growth (1559 [25·9%] of 6030). Breastfeeding at discharge was reported in 2784 (46·2%) of 6030 babies and this proportion increased during the study period. Among babies that suckled at the breast, the first breastfeed was at a median age of 7 days (IQR 6–9). Among babies that were fed maternal breastmilk (including by bottle or gastric tube), median age at first receiving maternal breastmilk was 5 days (IQR 4–6). Survival to discharge was high (5444 [90·3%] of 6030). Median length of stay in the neonatal unit was 11 days (IQR 8–16). Most babies (5640 [93·5%] of 6030) had a central line placed, for a median duration of 5 days (IQR 3–6). 1208 (20·0%) of 6030 babies had an episode of hypoglycaemia recorded during their neonatal stay.

**Figure 1:**
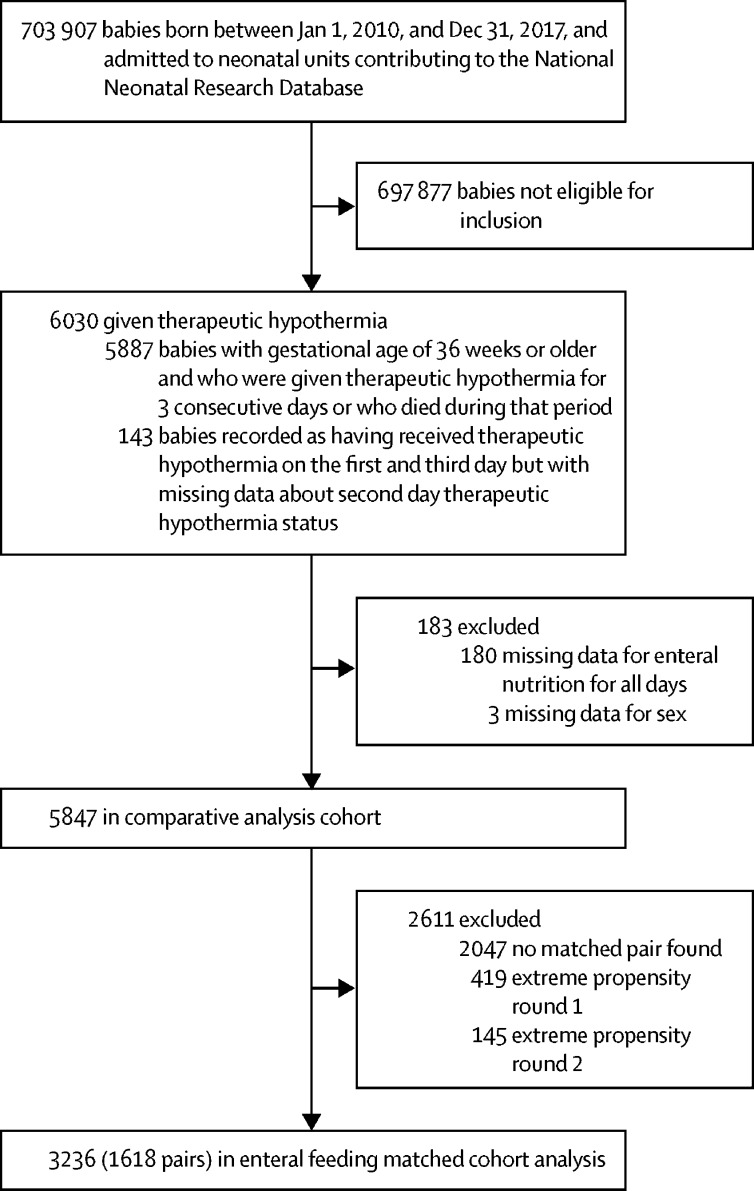
Participant flow for the primary analysis For a definition of extreme propensity see the appendix (p 10).

**Figure 2:**
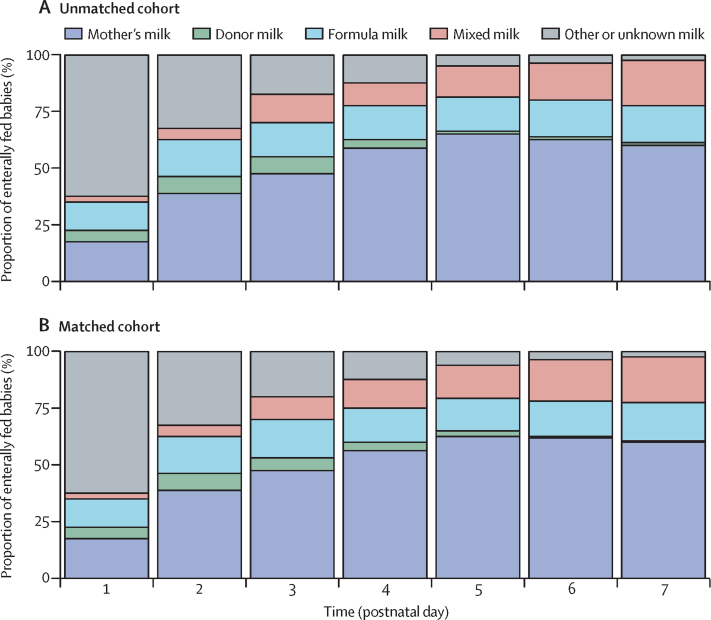
Types of milk used for enteral feeding during therapeutic hypothermia

For comparative analyses, we formed a matched cohort of 3236 babies (1618 pairs; figure 1), and a good balance was achieved for all recorded background variables (table 1; appendix p 13). The incidence of severe necrotising enterocolitis was very low and comparative analyses were not done (table 2). After matching, pragmatically defined necrotising enterocolitis incidence was lower among babies fed during therapeutic hypothermia than in babies who were not fed (difference −0·5%, 95% CI −1·0 to −0·1; p=0·028; table 3). The number of culture-positive late-onset infections was similar between groups; but pragmatically defined late-onset infection was less common in babies who were fed than not fed. Survival to discharge was higher in babies that were fed than were not fed; as was breastfeeding at discharge). Incidence was similar between groups for recorded hypoglycaemia and weight-for-gestational-age SD score at discharge from neonatal unit. First breastfeed and first breastmilk feed were earlier in babies that were fed than unfed during therapeutic hypothermia. Length of neonatal unit stay, duration of parenteral nutrition, and days with a central line in situ were all lower in babies who were fed than unfed (Table 2, Table 3). These findings were robust to sensitivity analyses detailed in the appendix (p 14). If enteral feeds were given during therapeutic hypothermia, these were most commonly maternal breastmilk (figure 2).

**Table 1:** Background variables by feeding group for unmatched and matched cohorts of babies receiving therapeutic hypothermia

		**Unmatched cohort**		**Matched cohort**	
		No enteral feeds (n=3975)	Enterally fed (n=1872)	No enteral feeds (n=1618)	Enterally fed (n=1618)
Sex					
	Male	2173 (54·7%)	1055 (56·4%)	903 (55·8%)	903 (55·8%)
	Female	1802 (45·3%)	817 (43·6%)	715 (44·2%)	715 (44·2%)
Gestational age at birth, weeks		39·3 (1·6)	39·5 (1·5)	39·5 (1·5)	39·5 (1·5)
Birthweight, g		3358 (621)	3395 (636)	3403 (635)	3386 (633)
Caesarean delivery		1921 (50·6%)	738 (41·2%)	649 (41·9%)	638 (41·2%)
	Missing	178	80	70	68
Median maternal age, years		30 (26–34)	31 (27–35)	31 (27–35)	31 (27–35)
Maternal chorioamnionitis suspected		406 (12·6%)	240 (15·2%)	202 (15·1%)	187 (13·6%)
	Missing	757	288	283	247
Maternal smoking during pregnancy		524 (15·4%)	191 (11·7%)	159 (11·3%)	169 (12·0%)
	Missing	566	244	207	205
Maternal race or ethnicity					
	White	2598 (77·5%)	1185 (76·5%)	1041 (77·1%)	1040 (77·3%)
	Asian or mixed Asian	399 (11·9%)	200 (12·9%)	174 (12·9%)	169 (12·6%)
	Black or mixed black African	277 (8·3%)	120 (7·7%)	99 (7·3%)	103 (7·7%)
	Other	80 (2·4%)	44 (2·8%)	36 (2·7%)	34 (2·5%)
	Missing	621	323	268	272
Maternal diabetes*		171 (4·3%)	75 (4·0%)	66 (4·1%)	60 (3·7%)
Maternal deprivation score†					
	Decile 1 or 2	1017 (29·4%)	351 (21·0%)	334 (27·0%)	311 (21·6%)
	Missing	515	204	379	374
Primiparous*		2107 (53·0%)	991 (52·9%)	857 (53·0%)	844 (52·2%)
Umbilical cord arterial pH					
	<6·9	913 (32·4%)	420 (31·1%)	368 (31·6%)	368 (31·6%)
	6·9–7·0	661 (23·5%)	318 (23·5%)	262 (22·5%)	262 (22·5%)
	>7·0	1240 (44·1%)	613 (45·4%)	536 (46·0%)	536 (46·0%)
	Missing	1161	521	452	452
Apgar score					
	0–1	695 (19·1%)	223 (12·8%)	231 (15·2%)	198 (13·1%)
	2–4	1476 (40·6%)	736 (42·1%)	650 (42·8%)	648 (42·8%)
	5–7	1140 (31·4%)	623 (35·6%)	490 (32·3%)	537 (35·4%)
	8–10	324 (8·9%)	166 (9·5%)	147 (9·7%)	132 (8·7%)
	Missing	340	124	100	103
Received chest compressions at resuscitation*		1555 (39·1%)	608 (32·5%)	560 (34·6%)	532 (32·9%)
Intubated at resuscitation*		2619 (65·9%)	1126 (60·1%)	1020 (63·0%)	995 (61·5%)
Mechanical ventilation on day of admission		3176 (83·1%)	1335 (73·4%)	1196 (77·6%)	1189 (75·3%)
	Missing	155	53	76	40
Treatment with inotropes on day of admission		1099 (29·0%)	320 (17·9%)	295 (19·3%)	288 (18·5%)
	Missing	180	80	90	63
Early onset, culture-positive blood infection*		44 (1·1%)	11 (0·6%)	26 (1·0%)	11 (0·7%)

Data are n (%), mean (SD), or median (IQR).

* Data were collected via ticking a box to indicate the presence of each condition and it is not possible to distinguish between missing data and a negative answer for boxes left empty.

† Maternal deprivation was calculated using the index of multiple deprivation decile based on the UK Office for National Statistics Lower-layer Super Output Area of the maternal residence.

**Table 2:** Outcomes by feeding group for unmatched and matched cohorts of babies receiving therapeutic hypothermia

	**Unmatched cohort**		**Matched cohort**	
	No enteral feeds (n=3975)	Enterally fed (n=1872)	No enteral feeds (n=1618)	Enterally fed (n=1618)
Severe necrotising enterocolitis (confirmed at surgery, post mortem, or recorded on death certificate)	<5	<5	<5	<5
Necrotising enterocolitis (pragmatic definition)	54 (1·4%)	11 (0·6%)	18 (1·1%)	9 (0·6%)
Late-onset, blood culture-positive infection	25 (0·6%)	5 (0·3%)	8 (0·5%)	<5
Late-onset infection (pragmatic definition)	1193 (30·0%)	321 (17·1%)	460 (28·4%)	271 (16·8%)
Survival at discharge	3498 (88·1%)	1794 (95·9%)	1465 (90·6%)	1552 (96·0%)
Hypoglycaemia	846 (21·3%)	316 (16·9%)	293 (18·1%)	269 (16·6%)
Onset of breastfeeding, days*	7 (6–10)	6 (5–8)	7 (6–9)	6 (5–8)
Breastfeeding at discharge*	1690 (42·5%)	1029 (55·0%)	752 (46·5%)	883 (54·6%)
Time to first maternal milk, days†	5 (5–6)	3 (2–4)	5 (5–6)	3 (2–4)
Received parenteral nutrition	1689 (42·5%)	683 (36·5%)	674 (41·6%)	596 (36·8%)
Duration of parenteral nutrition, days	3 (2–5)	3 (2–3)	3 (2–5)	3 (2–3)
Had a central venous line	3832 (96·4%)	1637 (87·4%)	1546 (95·5%)	1417 (87·6%)
Central venous line in situ, days	5 (4–7)	4 (3–5)	5 (4–6)	4 (3–5)
Weight Z score at discharge	−0·7 (−1·5 to 0·1)	−0·6 (−1·3 to 0·2)	−0·6 (−1·4 to 0·2)	−0·7 (−1·4 to 0·1)
Length of stay, days	11 (8–17)	10 (7–13)	11 (8–16)	10 (7–13)

Data are n (%) or median (IQR).

* Breastfeeding refers to suckling at the breast and does not include babies that received expressed breastmilk by bottle.

† First maternal milk includes receipt of maternal breastmilk by bottle.

**Table 3:** Outcomes for babies fed enterally versus not fed enterally during therapeutic hypothermia

	**No enteral feeds (n=1618)**	**Enterally fed (n=1618)**	**Difference between groups**	**Estimated odds ratio**	**p value**
Necrotising enterocolitis (pragmatic definition)	1·1% (0·7–1·4)	0·5% (0·2–0·9)	−0·5% (−1·0 to −0·1)	0·50 (0·22–1·12)	0·028
Late-onset infection (National Neonatal Audit Programme definition)	0·5% (0·2–0·7)	0·3% (0·04–0·4)	−0·2% (−0·5 to 0·1)	0·55 (0·17–1·80)	0·19
Late-onset infection (pragmatic definition)	28·4% (26·7–30·0)	16·7% (15·0–184)	−11·6% (−14·0 to −9·3)	0·51 (0·43–0·60)	<0·0001*
Hypoglycaemia	18·1% (16·7–19·5)	16·6% (15·0–18·3)	−1·5% (−3·7 to 0·6)	0·90 (0·75–1·08)	0·17
Survival at discharge	90·8% (89·7–91·8)	96·0% (95·0–96·8)	5·2% (3·9–6·6)	2·42 (1·80–3·26)	<0·0001*
Breastfeeding at discharge	46·7% (44·8–48·5)	54·6% (52·4–56·8)	8·0% (5·1–10·8)	1·38 (1·20–1·58)	<0·0001*
Length of stay, days	14·8 (14·2–15·5)	12·7 (12·0–13·3)	−2·2 (−3·0 to −1·2)	NA	<0·0001*
Onset of breastfeeding, days†	8·7 (8·4–9·0)	7·3 (6·9–7·7)	−1·4 (−1·9 to −0·9)	NA	<0·0001*
First maternal milk, days‡	5·4 (5·4–5·5)	3·3 (3·2–3·4)	−2·1 (−2·2 to −2·0)	NA	<0·0001*
Duration of parenteral nutrition, days	3·7 (3·5–3·8)	3·0 (2·7–3·4)	−0·7 (−1·1 to −0·2)	NA	0·0018*
Duration of central venous line, days	5·5 (5·3–5·7)	4·3 (4·1–4·5)	−1·2 (−1·5 to −0·9)	NA	<0·0001*
Weight-for-gestational-age SD score at discharge from neonatal unit	−0·60 (−0·65 to −0·55)	−0·54 (−0·59 to −0·48)	0·06 (−0·01 to −0·13)	NA	0·11

Data are estimated odds ratio (95% CI). Results were averaged over the 25 replications of the matching procedure.

* Outcome measure remains statistically significant after accounting for multiple testing using the Bonferroni correction. NA=odds ratio could not be estimated for continuous data.

† Breastfeeding refers to suckling at the breast and does not include babies that received expressed breastmilk by bottle.

‡ First maternal milk includes receipt of maternal breastmilk by bottle.

## Discussion

In this large, population-based cohort of term and near-term infants treated with therapeutic hypothermia, we identified variation in nutritional practice, with sizable minorities of babies receiving milk feeds during hypothermia. We showed that severe necrotising enterocolitis (using a robust and validated definition) is rare in this population, with an incidence of approximately one per 1000 babies. Late-onset culture-positive bloodstream infection is also rare, occurring in approximately five per 1000 infants, although treatment for presumed infection is common.

Almost one in three babies who receive therapeutic hypothermia in NHS neonatal units are fed during hypothermia, predominantly with maternal breastmilk. Necrotising enterocolitis is rare in these babies, and after matching for an extensive list of background characteristics, pragmatically defined necrotising enterocolitis was diagnosed less frequently in babies fed during therapeutic hypothermia than in babies who were unfed. Milk feeding during therapeutic hypothermia was also associated with other beneficial outcomes, including shorter length of neonatal unit stay, higher incidence of breastfeeding, and a lower incidence of suspected infection, all after matching for multiple potential confounding factors.

To our knowledge, this study assesses the largest and most comprehensive cohort of babies who have received therapeutic hypothermia. Necrotising enterocolitis is well described in case studies and single-centre series in babies with hypoxic ischaemic encephalopathy;^21^, ^22^ however, there has been a paucity of robust incidence data. By applying a validated definition of severe necrotising enterocolitis with confirmation of cases to large-scale population data,^23^ we have for the first time produced robust incidence rates for necrotising enterocolitis in babies receiving therapeutic hypothermia. Our finding that enteral feeding during therapeutic hypothermia is safe and might be associated with benefit is supported by the few data from previous studies. A small, retrospective case-control study^5^ of 34 infants compared minimal enteral nutrition with withholding feeds during therapeutic hypothermia, and found that duration of parenteral nutrition and hospital stay were lower in the enteral feeding group, although the study did not adjust for illness severity, which differed between groups. Another small retrospective study compared 34 babies cared for in the UK, which commonly withheld feeds, versus 51 babies cared for in Sweden, which commonly initiated feeds during hypothermia.^6^ In keeping with our data, the study found no complications in either group and a higher proportion of babies breastfeeding at discharge among the babies in Sweden. Our study adds considerably to the existing literature through the large, population-level nature of the cohort and by application of statistically robust approaches to deal with potential confounding in comparative analyses.

We aimed to identify optimal approaches to nutrition during therapeutic hypothermia, through comparative analyses of babies fed milk and those not fed milk. Although we did not undertake a randomised trial, we applied multiple approaches to reduce bias. We used a comprehensive range of background data items to form matched groups, balanced on all measured potential confounders. We followed a detailed, preregistered protocol that specified exposures, background factors, outcomes and the data items used to define outcomes, and the matching process.^14^ Also, the use of routinely recorded data reduced the risk of ascertainment bias because data collection occurred far in advance of study conception. Furthermore, we applied sensitivity analyses to see whether data quality and definitions of the nutritional exposures influenced results. By using existing data, we were able to include a large number of infants and to examine even rare outcomes, such as pragmatically defined necrotising enterocolitis and culture-positive bloodstream infection; the sample size was several times larger than all previous randomised trials of therapeutic hypothermia combined.

The most important limitation of this study stems from the non-randomised study design used; the matched approach that was applied was only able to account for measured confounders. Although we used a wide range of background and day 1 clinical data items to form matched groups, and the statistical measures of balance indicated that an acceptable balance had been obtained, we cannot exclude the chance that factors might have differed by small but clinically relevant degrees between the groups, or that there were important differences in unmeasured factors between the comparator groups. In matched analyses, we found higher survival to neonatal unit discharge in babies that were fed than were unfed during therapeutic hypothermia; this difference in survival is unlikely to be caused by enteral feeding during therapeutic hypothermia and suggests that residual confounding is present in the enteral comparison. Furthermore, there might be other additional markers of sickness that are discernible to clinicians and which influence decision making regarding enteral feeding, but which are not captured, even in the extensive data items held in the NNRD. Such residual and unmeasured confounding by indication might overestimate the benefit associated with enteral feeding, and results should be interpreted accordingly. Another limitation of this study is the smaller than planned number of infant pairs; however, the matched groups were still considerably larger than previous trials and had power to detect small but clinically relevant differences of 1·0% in necrotising enterocolitis. Although we had broad parent involvement in the selection of outcomes for this study, we were limited (by the data source used) to short-term outcomes, which might not completely reflect the outcomes of greatest importance to parents and patients.^13^ As with any study that uses routinely recorded data, data completeness and accuracy are dependent on the health-care professionals who enter the data, and might vary between sites. Specifically, data describing daily volume of feeds are highly incomplete, therefore we were unable to describe how much enteral feed was given or how quickly feeds were increased. Furthermore, culture-positive bloodstream infection is likely to be under-reported in systems such as the NNRD that use routinely recorded UK neonatal clinical data,^17^ hence our a-priori analysis used a pragmatic definition of late-onset infection. The NNRD is formed from data entered as part of a baby's clinical care record; these data are used for multiple purposes including national audits, funding, and staffing, and data held in the NNRD have been validated against independent clinical trial data.^11^

Overall, necrotising enterocolitis is rare in term and near-term babies receiving therapeutic hypothermia and might be less common in babies who were fed during therapeutic hypothermia. The introduction of enteral feeds in term and near-term babies during therapeutic hypothermia appears to be safe and might be associated with benefits, including a higher proportion of babies breastfeeding at discharge, and shorter length of neonatal unit stay.

## Data sharing

The deidentified individual participant data that were used in this study are available subject to NNRD steering board approval of a data extract request, the necessary regulatory approvals, and a charge to cover costs. The NNRD data dictionary is available online.

## Declaration of interests

We declare no competing interests.
