# Supplementary material for: Feeding during neonatal therapeutic hypothermia, assessed using routinely collected National Neonatal Research Database data: a retrospective, UK population-based cohort study
Source: Lancet Child Adolesc Health. 2021 Jun;5(6):408–16. doi: 10.1016/S2352-4642(21)00026-2 (PMC8131202; doi:10.1016/S2352-4642(21)00026-2)
Supplement: Supplementary appendix [file mmc1.pdf]

THE LANCET
Child & Adolescent Health

Supplementary appendix

This appendix formed part of the original submission and has been peer reviewed. We post it as supplied by the authors.

Supplement to: Gale C, Longford NT, Jeyakumaran D, et al. Feeding during neonatal therapeutic hypothermia, assessed using routinely collected National Neonatal Research Database data: a retrospective, UK population-based cohort study. *Lancet Child Adolesc Health* 2021; published online April 20. http://dx.doi.org/10.1016/S2352-4642(21)00026-2.

Supplementary material
Feeding during neonatal therapeutic hypothermia: a population level observational study using routinely collected data held in the National Neonatal Research Database

Contents

	page
**Study Steering Committee members**	1
**Supplemental table 1: Extraction procedures and definitions of enteral feeding exposure variable**	2
**Supplemental table 2: Extraction procedures, definitions and classifications of background variables**	2
**Supplemental table 3: Extraction procedures and definitions of outcome variables**	6
**Statistical methods: Propensity modelling**	9
**Statistical methods: Matching on propensity scores**	9
**Statistical methods: Assessment of the quality of the match**	10
**Supplemental figure 1: Histograms of estimated propensity scores**	11
**Supplemental figure 2: Balance plot (1:1 matching within propensity score deciles)**	12
**Supplemental table 4: Estimates of the effect of receiving enteral feeds for binary and continuous outcomes from sensitivity analyses**	13
**United Kingdom Neonatal Collaborative leads at contributing neonatal units**	15

Study Steering Committee members

David Odd (independent Chair), Senior Clinical Lecturer in Neonatal Medicine and Consultant Neonatologist, University of Bristol and North Bristol NHS Trust

Louise Linsell (independent), Senior Medical Statistician, Clinical Trials Unit, National Perinatal Epidemiology Unit (NPEU), University of Oxford

James Carpenter (independent), Professor of Medical Statistics, London School of Hygiene and Tropical Medicine

Carol Rhodes (independent), Mother of child who received therapeutic hypothermia as a baby

Chris Gale (non-independent), Reader in Neonatal Medicine and Consultant Neonatologist, Imperial College London and Chelsea and Westminster NHS Foundation Trust

Supplemental table 1: Extraction procedures and definitions of enteral feeding exposure variable

Variable	Data items from NNRD	Definition
Enteral Feeding	ENTERAL FEEDING GROUP DEFINED AS<br>Any of the following items entered in the *Daily Care Fluids and Feeding* during the first 3 days<br>• Any entry (1-6) under ENTERAL FEED TYPE GIVEN<br>• OR any entry (0-88) under FORMULA MILK OR MILK FORTIFIER TYPE<br>• OR any value >0 for TOTAL VOLUME OF MILK RECEIVED<br>• OR any entry (1-8) under ENTERAL FEEDING METHOD<br><br>NO ENTERAL FEEDING GROUP DEFINED AS<br>All other babies not fulfilling above criteria	Dichotomous (No enteral feeds=0; provided enteral feeds=1)

Supplemental table 2: Extraction procedures, definitions and classifications of background variables

Variable	Data items from NNRD	Definition(s)	Classification
Cord blood gas pH in bands	*Labour and Delivery Details*<br>UMBILICAL CORD BLOOD pH LEVEL (ARTERIAL)<br>*Or, if not recorded, use*<br>*Labour and Delivery Details*<br>UMBILICAL CORD BLOOD pH LEVEL (ARTERIAL)	**CordpHArt:** Tricotomised into bands: >7·0, 6·9-7·0, <6·9	Principal
Birth year	*Baby Demographics* YEAR AND MONTH OF BIRTH (BABY)	**BirthYear:** Categorised into two-year bands: 2010-2011, 2012-2013, 2014-2015, 2016-2017	Principal
Gestational age week	*Baby Demographics* GESTATION LENGTH (AT DELIVERY):<br>Gestational weeks and days	**GAweeks:** Integers	Highly important
Birthweight	*Baby Demographics* BIRTH WEIGHT	**Bweight:** Original entries trimmed from above at 3500g and entries smaller than 1000g with a non-zero digit are multiplied by 10. Square-root transformed.	Highly important
Sex	*Baby Demographics* PERSON PHENOTYPIC SEX	**Sex:** Dichotomous (Male=0; Female=1)	Highly important
Emergency resuscitation drugs administered	*Labour and Delivery Details*<br>NEONATAL RESUSCITATION METHOD Dichotomous:<br>Y= code 17 (Adrenaline) OR 88 (any other drug)<br>N= any other codes OR no code	**ResusDrugs:** Dichotomous (No=0; Yes=1)	Highly important
Instrument of delivery	*Labour and Delivery Details*<br>DELIVERY INSTRUMENT TYPE	**InstrDeliv:** Dichotomised (No instrument used=0; Forceps or ventouse=1)	Highly important
Mode of delivery	*Labour and Delivery Details* MODE OF DELIVERY<br>Categorical: codes=1-4<br>AND<br>*Labour and Delivery Details IN LABOUR BEFORE CAESARIAN SECTION INDICATOR*=Y/N	**Delivery:** Dichotomous (Vaginal=0, Caesarean=1)	Highly important
Maternal smoking status	*Pregnancy Details* MOTHER CURRENT SMOKER AT BOOKING INDICATOR<br>(categorical, codes 1-6)	**SmokePreg:** Dichotomised (Not smoking=0; Smoking during pregnancy=1)<br><br>**SmokePregMs:** Binary missing indicator created (Not missing=0; Missing=1)	Highly important
Maternal suspected chorioamnionitis	*Labour and Delivery Details*<br>INTRAPARTUM ANTIBIOTICS GIVEN INDICATORS	**IntrPartAntiB:** Dichotomous (No intrapartum antibiotics given=0, Intrapartum antibiotics=1)	Highly important
Apgar score at 1 minute	*Labour and Delivery Details* APGAR SCORE (1 MINUTE)<br>Continuous: 0-10	**APGAR1min:** Categorical (0-10)<br><br>**APGAR1minMs:** Binary missing indicator created (Not missing=0; Missing=1)	Highly important
Apgar score at 5 minutes	*Labour and Delivery Details* APGAR SCORE (5 MINUTE)	**APGAR5min:** Categorical (0-10)	Highly important

Supplemental table 2: Extraction procedures, definitions and classifications of background variables

Variable	Data items from NNRD	Definition(s)	Classification
	Continuous: 0-10	**APGAR5minMs:** Binary missing indicator created (Not missing=0; Missing=1)	
Umbilical cord base excess	*Labour and Delivery Details* UMBILICAL CORD BLOOD BASE EXCESS CONCENTRATION (ARTERIAL)<br>Continuous<br>OR if not available use<br>*Labour and Delivery Details* UMBILICAL CORD BLOOD BASE EXCESS CONCENTRATION (VENOUS)	**CordBaseExcess:** Continuous (to 1 decimal place)<br><br>**CordBaseExcessMs:** Binary missing indicator created (Not missing=0; Missing=1)	Highly important
Admission mean blood pressure	*Admission Details* MEAN ARTERIAL BLOOD PRESSURE (ON ADMISSION TO NEONATAL CRITICAL CARE)<br>Continuous	**AdmitBP:** Continuous, square-root transformed<br><br>**AdmitBP.Ms:** Binary missing indicator created (Not missing=0; Missing=1)	Highly important
Admission blood glucose	*Admission Details* BLOOD GLUCOSE CONCENTRATION (ON ADMISSION TO NEONATAL CRITICAL CARE)<br>Continuous	**AdmitBG:** Continuous, trimmed from above at 20<br><br>**AdmitBG.MS:** Binary missing indicator created (Not missing=0; Missing=1)	Highly important
Admission oxygen saturation	*Admission Details* OXYGEN SATURATION (ON ADMISSION TO NEONATAL CRITICAL CARE)<br>Continuous	**AdmitOS:** Continuous, trimmed to be within the range (50, 100)<br><br>**AdmitOS.Ms:** Binary missing indicator created (Not missing=0; Missing=1)	Highly important
Maternal deprivation score (from lower super output area)	*Parents Demographics* POSTCODE OF USUAL ADDRESS (LSOA)	**LSOAdec:** Categorised into deciles (1, 2,…, 10)<br><br>**LSOAdecMs:** Binary missing indicator created (Not missing=0; Missing=1)	Highly important
Multiplicity	*Labour and Delivery Details* BIRTH ORDER (MATERNITY SERVICES)<br>*Labour and Delivery Details* NUMBER OF FETUSES (NOTED DURING PREGNANCY EPISODE)	**MultipleBrt:** Dichotomised (single birth=0; multiple births=1)	Moderately important
Maternal age	*Parents Demographics* YEAR OF BIRTH (MOTHER)	**MaternalAge:** Continuous (years) trimmed to be within range 17- 45	Moderately important
Maternal duration of rupture of membranes (time in hours)	*Labour and Delivery Details* RUPTURE OF MEMBRANES DATE TIME *or* RUPTURE OF MEMBRANES YEAR AND MONTH and<br>*NUMBER OF MINUTES (BIRTH TO EVENT)*<br>(continuous)		*Variable not used. (Too many missing values)*
Maternal disease during pregnancy	*Labour and Delivery Details* SIGNIFICANT MATERNAL PYREXIA IN LABOUR INDICATOR (Y/N)<br><br>*Pregnancy Details* MATERNITY COMPLICATING MEDICAL DIAGNOSIS TYPE<br>Dichotomous: Y=code 16 (endocrine disorder), N=any other or no code<br><br>*Pregnancy Details* MATERNITY COMPLICATING MEDICAL DIAGNOSIS TYPE<br>Dichotomous: Y=code 08 (diabetes)<br>OR<br>*Pregnancy Details* MATERNITY OBSTETRIC DIAGNOSIS TYPE<br>Dichotomous: Y=code 06 (gestational diabetes mellitus)	**MaternalDis:** Dichotomised (No diagnosis=0; At least one of pyrexia in labour, hypothyroid, diabetes=1)	Moderately important

Supplemental table 2: Extraction procedures, definitions and classifications of background variables

Variable	Data items from NNRD	Definition(s)	Classification
	N=any other or no code		
Maternal ethnicity	*Parents Demographics* ETHNIC CATEGORY (MOTHER) (categorical)<br>Coded as:<br>WHITE (A - British, B - Irish, C - Any other white background);<br>MIXED (D - White and Black Caribbean. E - White and Black African, F - White and Asian, G - Any other mixed background);<br>ASIAN OR ASIAN BRITISH (H - Indian, J - Pakistani, K - Bangladeshi, L - Any other Asian Background);<br>BLACK OR BLACK BRITISH (M - Caribbean, N - African, P - Any other Black background);<br>OTHER ETHNIC GROUPS (R - Chinese, S - Any other ethnic group);<br>UNKNOWN (Z, DTA - Not stated, 99 - Not known)<br><br>This data item is based on self-reported ethnicity as recorded in maternity notes	**Ethnicity:** Categorised into four groups (White=1; Asian & Mixed=2; Black & Mixed=3; Other and not given=4)	Moderately important
Parity of mother (primiparous Y/N)	*Pregnancy Details* PREGNANCY TOTAL PREVIOUS PREGNANCIES<br>Dichotomous: code 00=Y; code 01-29=N	**Primiparous:** Dichotomous (Not first pregnancy=0; First pregnancy=1)	Moderately important
Chest compressions administered	*Labour and Delivery Details* NEONATAL RESUSCITATION METHOD<br>Dichotomous: Code 16=Y; any other code=N	**ChestCompr:** Dichotomous (No chest compressions applied=0; Chest compressions applied=1)	Moderately important
Intubated at resuscitation	*Labour and Delivery Details* NEONATAL RESUSCITATION METHOD<br>Dichotomous: Code 15=Y; any other code=N	**Intubation:** Dichotomous (Not intubated=0; Intubated=1)	Moderately important
Time to first spontaneous breath	*Labour and Delivery Details* TIME BETWEEN DELIVERY AND SPONTANEOUS RESPIRATION CODE<br>Continuous	**SpontRespTime:** Dichotomised (≤5 mins=0; >5 mins=1)<br><br>**SpontRespTimeMs:** Binary missing indicator created (Not missing=0; Missing=1)	Moderately important
Admission heart rate	*Admission Details* HEART RATE (ON ADMISSION TO NEONATAL CRITICAL CARE)<br>Continuous	**AdmitHR:** Continuous, trimmed to be within the range 80-100<br><br>**AdmitHR.Ms:** Binary missing indicator created (Not missing=0; Missing=1)	Moderately important
Admission temperature	*Admission Details* TEMPERATURE (ON ADMISSION TO NEONATAL CRITICAL CARE)<br>Continuous	**AdmitTempCe:** Continuous, trimmed to be within 26-40<br><br>**AdmitTempMs:** Binary missing indicator created (Not missing=0; Missing=1)	Moderately important
Positive blood or cerebrospinal fluid culture with a recognised pathogen recorded in the first 3 days	Defined from *Infection Cultures (Episodic)* recorded up to and including day 3<br>• Pure growth of pathogen from blood<br>OR<br>• Pure growth of pathogen from CSF	**Infection:** Dichotomous (0=No infection; 1=infection)	Moderately important
Treatment for low blood pressure with an intravenous inotrope (e.g. dopamine, noradrenaline)	*Daily Care Medication* on day 1 only<br>• 500098 Dopamine<br>• 500096 Dobutamine	**Inotropes:** Dichotomous (Inotropes not administered=0; Inotropes administered=1)	Moderately important

Supplemental table 2: Extraction procedures, definitions and classifications of background variables

Variable	Data items from NNRD	Definition(s)	Classification
	• 500056 Adrenaline<br>• 500210 Noradrenaline<br>• 500116 Hydrocortisone<br>• 1010173 Milrinone<br>Dichotomous: any of above=Y, none of above=N<br>OR<br>*Daily Care Cardiovascular* INOTROPE INFUSION RECEIVED INDICATOR Y/N		
Mechanical ventilation method	*Daily Care Respiratory* on day 1 only; RESPIRATORY SUPPORT MODE<br>Dichotomous: Codes 1, 2, 3=Y; any other or no code =N	**RespiSupprt:** Dichotomous (Respiratory support not provided=0; Respiratory support provided=1)	Moderately important
Received inhaled nitric oxide (Y/N)	*Daily Care Respiratory* on day 1 only; NITRIC OXIDE GIVEN INDICATOR<br>Dichotomous: Y/N	**NitricOxide:** Dichotomous (Nitric oxide not given=0; Nitric oxide given=1)	Moderately important
Required acute postnatal transfer, within 24 hours (Y/N)	*Admission Details* SITE CODE (OF ADMITTING NEONATAL UNIT) or ORGANISATION CODE (OF ADMITTING NEONATAL UNIT)<br>Different from<br>*Baby Demographics* SITE CODE (OF ACTUAL PLACE OF DELIVERY) or ORGANISATION CODE (OF ACTUAL PLACE OF DELIVERY)<br>And<br>*Baby Demographics* EPISODE NUMBER	**PostNTransfer:** Dichotomous (No transfer=0; Transfer=1)	Moderately important
Maternal occupation	*Parents Demographics (withheld)* OCCUPATION MOTHER (SNOMED CT)	**MumJob:** Dichotomous (No occupation=0; Any occupation=1)	Moderately important
Onset of labour	*Labour and Delivery Details* LABOUR OR DELIVERY ONSET METHOD CODE	**OnsetLabour:** Categorised into four groups (Not in labour=0; Spontaneous=1; Induced=2; Missing=9)	Moderately important
Time to admission	*Admission Details* CRITICAL CARE START YEAR AND MONTH and NUMBER OF MINUTES (BIRTH TO EVENT)	**AdmitTime:** log-transformed with zero recoded to zero	Moderately important
Presentation at delivery	*Labour and Delivery Details* PRESENTATION AT DELIVERY<br>1 - Breech<br>2 - Cephalic<br>3 - Transverse<br>8 - Other<br>9 - Unknown	**FetusAtDelivC:** Dichotomised (Cephalic=1, Not cephalic=0)	Moderately important
Blood transfusion	*Daily care blood transfusion* BLOOD TRANSFUSION PRODUCT TYPE on day 1 only	**BloodTrans:** Dichotomised (No=0; Yes=1)	Moderately important
Maternal or obstetric medical problem	*Pregnancy Details* MATERNITY OBSTETRIC DIAGNOSIS TYPE (CURRENT PREGNANCY)<br><br>*Pregnancy Details MATERNITY MEDICAL DIAGNOSIS TYPE (CURRENT PREGNANCY)*	**ProblMedic:** Dichotomised (No medical problems=0; Some medical problems=1)	Moderately important

Supplemental table 3: Extraction procedures and definitions of outcome variables

Variable	Data items from NNRD	Definition
Severe NEC	Gestational age specific NEC score based on Battersby et al., JAMA Pediatrics, 2017. Data items needed:<br>ABDOMINAL X-RAYS (EPISODIC)<br>• CONDITION SEEN IN ABDOMEN DURING X-RAY (NNRD field ID: XRayAppearances)<br>• ABDOMINAL X-RAY PERFORMED REASON (NNRD field ID: ClinicalFindings)<br>• TRANSFERRED FROM NEONATAL INTENSIVE CARE UNIT FOR NECROTISING ENTEROCOLITIS MANAGEMENT INDICATOR (NNRD field ID: TransferredForFurtheManagement)<br>• LAPAROTOMY FOR NECROTISING ENTEROCOLITIS INDICATION CODE<br>• NEC CONFIRMED BY VISUAL INSPECTION DURING LAPAROTOMY (INDICATOR)HISTOLOGY CONFIRMED NECROTISING ENTEROCOLITIS FOLLOWING LAPAROTOMY INDICATOR<br>• POSTMORTEM CONFIRMED NEC<br>• CAUSE OF DEATH<br>Only available following introduction of ABDOMINAL X-RAY (EPISODIC) field<br><br>Cases identified using these data items were individually confirmed with clinicians.	Dichotomous (No severe NEC=0, Severe NEC=1)
Necrotising enterocolitis (non-UKNC definition)	The following entered in the *Daily Care Gastrointestinal* on any one day during stay in a neonatal unit<br>• Any entry (1 or 2) for TREATMENT TYPE FOR NECROTISING ENTEROCOLITIS<br>OR the following diagnostic codes<br>• 1010683 *Necrotising enterocolitis – suspected*<br>• 10708 *Necrotising enterocolitis – Perforated*<br>• 15809 *Necrotizing enterocolitis*<br><br>AND<br>5 or more days nil by mouth defined by the *Daily Care Fluids and Feeding* for a continuous period of 5 days<br>• No under ENTERAL FEED TYPE GIVEN<br>• No entry under FORMULA MILK OR MILK FORTIFIER TYPE<br>• No value OR 0 for TOTAL VOLUME OF MILK RECEIVED<br>• No entry under ENTERAL FEEDING METHOD<br><br>WHILE ALSO RECEIVING<br>5 or more days of antibiotics over the same 5 days as the baby was nil by mouth, defined as 5 consecutive days of any of the following<br>*Daily care medication*<br>• 1010155 Benzyl Penicillin<br>• 1010158 Augmentin<br>• 1010179 Flucloxicillin<br>• 500012 Flucloxacillin<br>• 500016 Gentamicin<br>• 500072 *Co-amoxiclav*<br>• 500086 Co-amoxiclav<br>• 500084 Ciprofloxacin<br>• 500029 Netilmicin<br>• 500002 Amikacin<br>• 500211 Tazocin<br>• 500023 Metronidazole<br>• 500040 Vancomycin<br>• 500007 Cefotaxime<br>• 500004 Ampicillin<br>• 500009 Cefuroxime<br>• 500008 Ceftazidime<br>• 500175 Ceftriaxone<br>• 500032 Piperacillin<br>• 500206 *Oflacillin*<br>• 500005 Azlocillin<br>• 1010171 Linezolid<br>• 1010271 *Cefalexin*	Dichotomous (No NEC=0, NEC=1)

Supplemental table 3: Extraction procedures and definitions of outcome variables

Variable	Data items from NNRD	Definition
	• 1010139 Amoxicillin<br>• 500070 Amoxicillin<br>• 500128 Meropenem<br>• 500118 Imepenem<br>• 500145 *Imipenem*<br>• 500069 Ambisome (Liposomal Amphotericin)<br>• 500003 Amphotericin<br>• 1010195 Amphotericin Liposomal	
Late onset blood stream infection NNAP definition	Defined from *Infection Cultures (Episodic)* recorded after day 3<br>• Pure growth of pathogen from blood<br>OR<br>• Pure growth of pathogen from CSF<br>OR<br>• Either a pure growth of a skin commensal or a mixed growth with ≥3 clinical signs at the time of blood sampling	Dichotomous (No infection=0, Infection=1)
Late onset infection, non-NNAP	5 consecutive days of antibiotic treatment defined as 5 consecutive days of any of the following (including in combination and changing during the 5 days) after day 3<br>*Daily care medication*<br>• 1010155 Benzyl Penicillin<br>• 1010158 Augmentin<br>• 1010179 Flucloxicillin<br>• 500012 Flucloxacillin<br>• 500016 Gentamicin<br>• 500072 *Co-amoxiclav*<br>• 500086 Co-amoxiclav<br>• 500084 Ciprofloxacin<br>• 500029 Netilmicin<br>• 500002 Amikacin<br>• 500211 Tazocin<br>• 500023 Metronidazole<br>• 500040 Vancomycin<br>• 500007 Cefotaxime<br>• 500004 Ampicillin<br>• 500009 Cefuroxime<br>• 500008 Ceftazidime<br>• 500175 Ceftriaxone<br>• 500032 Piperacillin<br>• 500206 *Oflacillin*<br>• 500005 Azlocillin<br>• 1010171 Linezolid<br>• 1010271 *Cefalexin*<br>• 1010139 Amoxicillin<br>• 500070 Amoxicillin<br>• 500128 Meropenem<br>• 500118 Imepenem<br>• 500145 *Imipenem*<br>• 500069 Ambisome (Liposomal Amphotericin)<br>• 500003 Amphotericin<br>• 1010195 Amphotericin Liposomal	Dichotomous (No infection=0, Infection=1)
Survival to discharge	Defined from the *Discharge Details* from final neonatal unit stay<br>• DISCHARGE DESTINATION FROM NEONATAL CRITICAL CARE = 1, 2, 4, 5, 6 (NOT code 3, *Died*)	Dichotomous (Died during neonatal stay=0, Survived until discharge =1)
Length of neonatal unit stay	Defined as the total number of days a baby received neonatal care (any level of care) from *Daily Care General Information* - LOCATIONS OF HIGHEST LEVEL OF CARE	Continuous, integers
Hypoglycaemia	Defined as any of the following *diagnostic codes* recorded at any time during an babies neonatal units stay:<br>• 15771 Iatrogenic neonatal hypoglycaemia<br>• 15773 Neonatal hypoglycaemia	Dichotomous (No hypoglycaemia=0, Hypoglycaemia =1)
Breastfeeding at discharge	Defined from final day of neonatal care entry in *Daily Care Fluids and Feeding* of<br>• ENTERAL FEED TYPE GIVEN = code 1 (Breastfeeding)<br>OR<br>• ENTERAL FEEDING METHOD = code 1 (breast)<br>Where final day is not entered, penultimate day will be used	Dichotomous (Not suckling at the breast at discharge=0, Suckling at the breast at discharge=1)

Supplemental table 3: Extraction procedures and definitions of outcome variables

Variable	Data items from NNRD	Definition
Onset of breastfeeding	Number of days until first entry in *Daily Care Fluids and Feeding* of<br>• ENTERAL FEED TYPE GIVEN = code 1 (Breastfeeding)<br>OR<br>• ENTERAL FEEDING METHOD = code 1 (breast)	Continuous, integers
Time to first maternal breast milk feed	First day on which a baby is recorded to be receiving maternal breast milk by any route (including suckling at the breast, by bottle or nasogastric tube) defined as *Daily Care Fluids and Feeding* of<br>• ENTERAL FEED TYPE GIVEN = code 1 (Breastfeeding); 2 (Mothers fresh expressed breast milk); 3 (Mothers frozen expressed breast milk); 4 (Donor expressed breast milk)<br>OR<br>• ENTERAL FEEDING METHOD = code 1 (breast)	Continuous, integers
Duration of parenteral nutrition	Defined as the number of days UNTIL a baby has:<br>• *Daily Care Fluids and Feeding* PARENTERAL NUTRITION RECEIVED INDICATOR = N<br>AND<br>• The following drug code NOT entered in the *Daily care medication:* 1010238 *Total parenteral nutrition*<br><br>This outcome will be analysed only for the ENTERAL FEEDING COMPARISON.	Continuous, integers
Number of days a baby has a central venous line in situ	Defined as the number of days that has a baby has:<br>• *Daily Care Fluids and Feeding* VASCULAR LINE TYPE IN SITU = code 3 (Umbilical venous line); 4 (Percutaneous central venous line ('long line'); 5 (Surgically inserted central venous line)	Continuous, integers
Weight SDS at discharge	Defined as the following data item on the final day of neonatal care:<br>• *Daily Care General Information* PERSON WEIGHT IN GRAMS<br>If final day is not entered, the penultimate day is used	Continuous

Statistical methods: Propensity modelling

We fitted a propensity model in which the observed intervention group as the outcome is related to the background variables. The outcome variable in propensity analysis is binary, so logistic regression is applied. Since we had many background (confounding) variables, a model had to be selected from multiple candidate models. We followed the step-wise approach proposed by Imbens and Rubin[1].

Background variables were reviewed by the Clinical Investigator Group (see Appendix 2). The background variables' relative clinical importance to the analysis was decided and variables were classified into three groups. These background variables were used to form matched groups for subsequent analysis.

The variables deemed to be of highest importance were termed principal background variables, of which there were two:

1. birth year
2. pH of arterial blood in the umbilical cord.

Highly important background variables are next in the hierarchy, of which there were 15:

1. birthweight (g)
2. gestational age (weeks)
3. sex
4. resuscitation drugs received
5. delivery instrument used (forceps or ventouse)
6. mode of delivery (vaginal or other)
7. smoking during pregnancy
8. suspected maternal chorioamnionitis
9. Apgar score at 1 minute
10. Apgar score at 5 minutes
11. umbilical cord blood base excess concentration (venous)
12. mean blood pressure at first neonatal unit admission
13. oxygen saturation at first neonatal unit admission
14. blood glucose concentration at first neonatal unit admission
15. maternal socioeconomic decile as defined using maternal lower-layer super output area (LSOA).

The remaining background variables are classified as moderately important and are listed in Supplemental Table 2. For variables with a considerable number of missing data, binary missing data indicators were also defined.

The background variables classified as being *highly important* were included in the model *a priori*. Models were then fitted with each of the remaining background variables added individually. The model with the largest value of the chi-squared statistic (with one degree of freedom) was adopted, if the test statistic exceeded 1·0. This procedure constitutes one cycle. In the next and following cycles, all the remaining background variables were tested similarly and the one with the largest value of the chi-squared statistic was retained. The cycles were stopped when none of the chi-squared statistics for including a covariate exceeded 1·0 and the variables included in the model at this point are referred to as the main effects.

Next interactions were selected for the propensity model. The main effects were sorted in descending order of their absolute t-ratios ($|estimate/st.error|$). For each variable A we formed a list of variables B for which the interaction $A \times B$ was an appropriate candidate for inclusion. For example, no two categories of a discrete variable could appear in an interaction. A continuous variable could be interacted with itself (the result is the quadratic transformation of the variable), but a binary variable could not. Similarly, a variable could not be interacted with its missing value indicator.

Starting with the first covariate we fitted the models with one interaction of this covariate added and selected up to two of the interactions that have the largest values of the chi-squared statistic for inclusion, subject to the condition that they exceeded 2.71 (the 10th percentile of the chi-squared distribution with 1 degree of freedom). When an interaction $A \times B$ was adopted (added to the model), the interaction $B \times A$ was removed from the list of candidate interactions, to avoid singularity in the model search that followed. After the interactions of the first covariate, the interactions of the second and successive covariates were tested and the model was expanded by the interactions found most important, subject to the constraint of including at most two interactions in each cycle.

The concluding model yielded the fitted propensities – the estimated probabilities of being assigned to the groups receiving enteral feeds, given each baby's background profile. Thus, each baby was associated with a (fitted) propensity. The set of babies in the analysis was then reduced by excluding babies with extreme propensities; first, by reducing to the subjects in the overlap of intervention and control group. That is, let the propensities in the two groups be in the respective ranges (m_1, M_1) and (m_2, M_2). Then all subjects with propensities smaller than $m = \max(m_1, m_2)$ and greater than $M = \min(M_1, M_2)$ were excluded. Another criterion, described in Imbens and Rubin[1], was applied to reduce the sampling variance of the average treatment effect to be evaluated. It yields a positive constant $\gamma < 1$. Subjects with propensities outside the range $(\gamma, 1 - \gamma)$ were discarded from the analysis. Such reduction of the dataset by discarding subjects with extreme propensities is referred to as trimming.

Next, the entire modelling exercise, with selection of the main effects (added to the covariates selected a priori) and selection of the interactions was repeated on the reduced (trimmed) dataset. This was followed by discarding subjects with extreme propensities (fitted by the revised model). Trimming was applied after each stage of model selection.

The variables in each final propensity model have no interpretation for inference. The sole purpose of the propensity model is to facilitate a good balance of all the background variables in matched groups.

Statistical methods: Matching on propensity scores

To form matched subgroups, we first formed background groups based on unique combinations of the two principal background variables. Four birth year groups (as birth year is grouped according to two-year bands) crossed with three cord blood pH groups generate 12 background groups. We then defined propensity groups within each background group by recoding the propensities to a set of (propensity) groups separated by cutpoints. An established method splits the propensities into K groups of approximately equal size. We use $K = 10$ to form propensity score deciles. Within each background group, a baby who received enteral feeds was paired to a randomly drawn baby that did not receive enteral feeds who fell within the same propensity group. After the matching process was complete, the matched pairs of babies were reconstituted as the intervention group (received enteral feeds) and control group (no enteral feeds) and termed the *matched cohort*.

Statistical methods: Assessment of the quality of the match

The selected (or any other) propensity model has no interpretation for inference; its sole purpose is to facilitate the formation of an exposure and control group (the matched cohorts) for the enteral feeding analysis that is well balanced with regard to measured background variables. It was essential that no outcome variables, or more precisely, no variables that have differing potential outcomes, were involved in this stage. The motivation for this is that the background should be considered in earnest, and that this is done with no fore-knowledge of the outcomes. Accordingly, assessing the balance on all the background variables is the only relevant diagnostic for the fitted propensities.

The imbalance of an ordinal variable across two groups is defined as the difference of the within-group means divided by the standard deviation pooled across the two groups. The absolute imbalance is defined as the absolute value of the imbalance. The imbalance for a set of ordinal variables is defined as the mean of the absolute imbalances of the variables. We used this statistic as a summary or characteristic of the (overall) imbalance of two (sub-) groups. Smaller values indicate tighter balance. Imbens and Rubin[1] regard the balance of a variable as satisfactory if its absolute imbalance is smaller than 0·1. For a dataset, original or formed by matching, we report the total of the absolute imbalances and the largest and smallest imbalances. Variables that are not ordinal, that is, categorical variables, are avoided by defining indicator (dummy) variables; $H - 1$ indicators for a variable with H categories. The choice of the 'omitted' (reference) category is immaterial.

Supplemental figure 1 presents histograms of the estimated propensity scores from the final enteral feeding propensity model by intervention (received enteral feeds) and control (no enteral feeds) groups. There is good overlap of the propensity scores in the exposure and control groups, so many matched pairs can be formed. Data from 574 babies (9·8% of total sample) were discarded due to extreme propensities.

Supplemental figure 2 presents the balance plot for the background variables included in the final enteral nutrition comparison propensity model. The dashed grey line indicates perfect balance between the enteral feeding groups for a specific background variable. The grey shaded area indicates the acceptable limits of imbalance for any variable, equivalent to an imbalance of $\leq 0 \cdot 1$ in absolute value. The imbalance for a specific background variable in the unmatched cohort is depicted by the bold dash and the light dash indicates the opposite of this imbalance (imbalance multiplied by -1), which represents the same extent of imbalance. The

imbalance in the matched cohort is marked by the black disc. The balances for the background variables are summarised by the mean of their absolute values. Prior to matching the mean balance is 0·064, and the balances are between -0·248 and 0·188. The mean balance for the matched dataset is 0·090, and the balances are between -0·037 and 0·029. The mean balances are displayed in Supplemental figure 2.

Supplemental figure 1: Histograms of estimated propensity scores. Thick vertical dashed lines indicate trimming thresholds for extreme propensities, thin vertical dashed lines indicate propensity deciles for babies retained for analysis

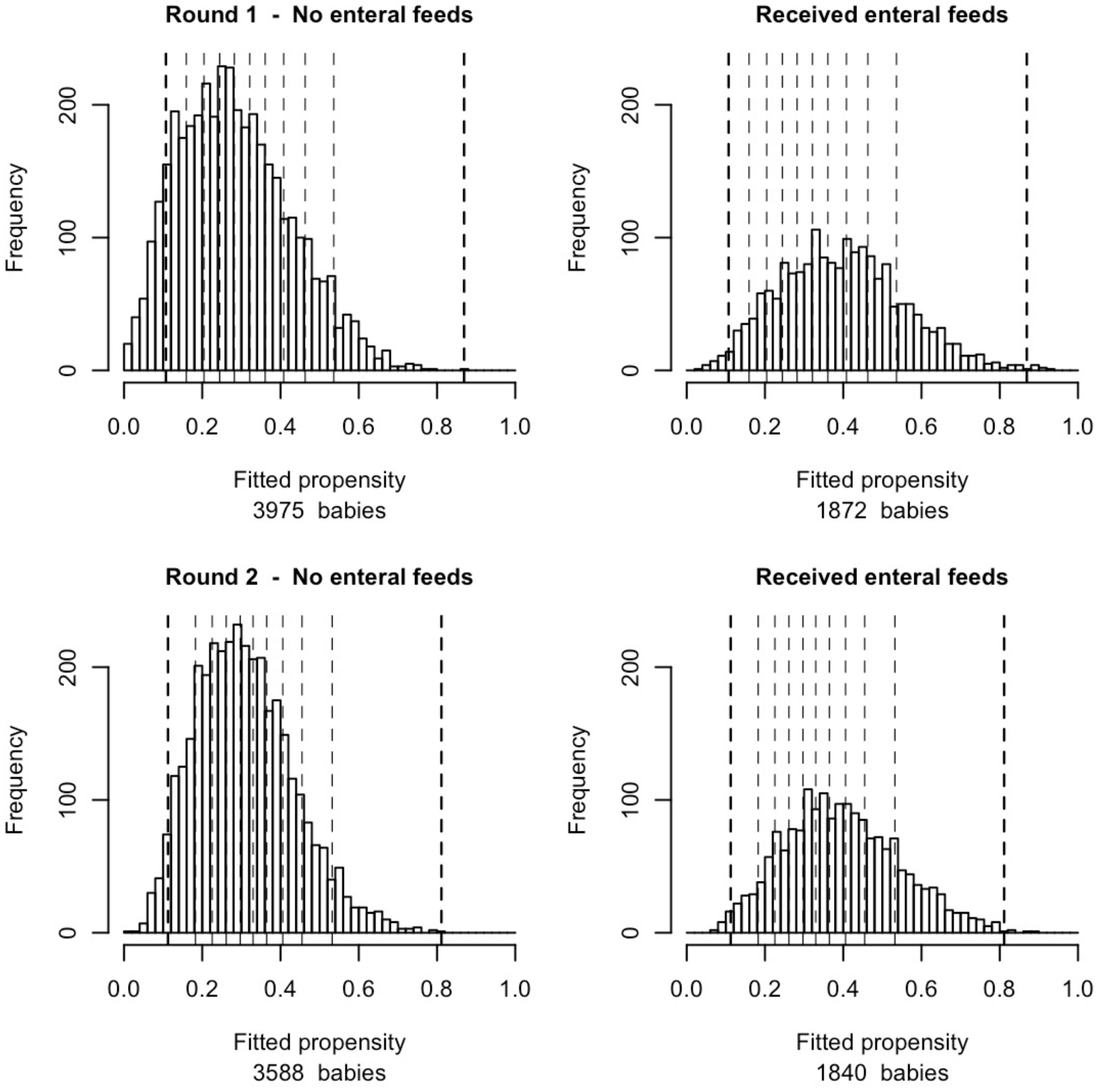

Supplemental figure 2: Balance plot (1:1 matching within propensity score deciles). The grey shaded area covers the region of acceptable balances, -0·1 to 0·1

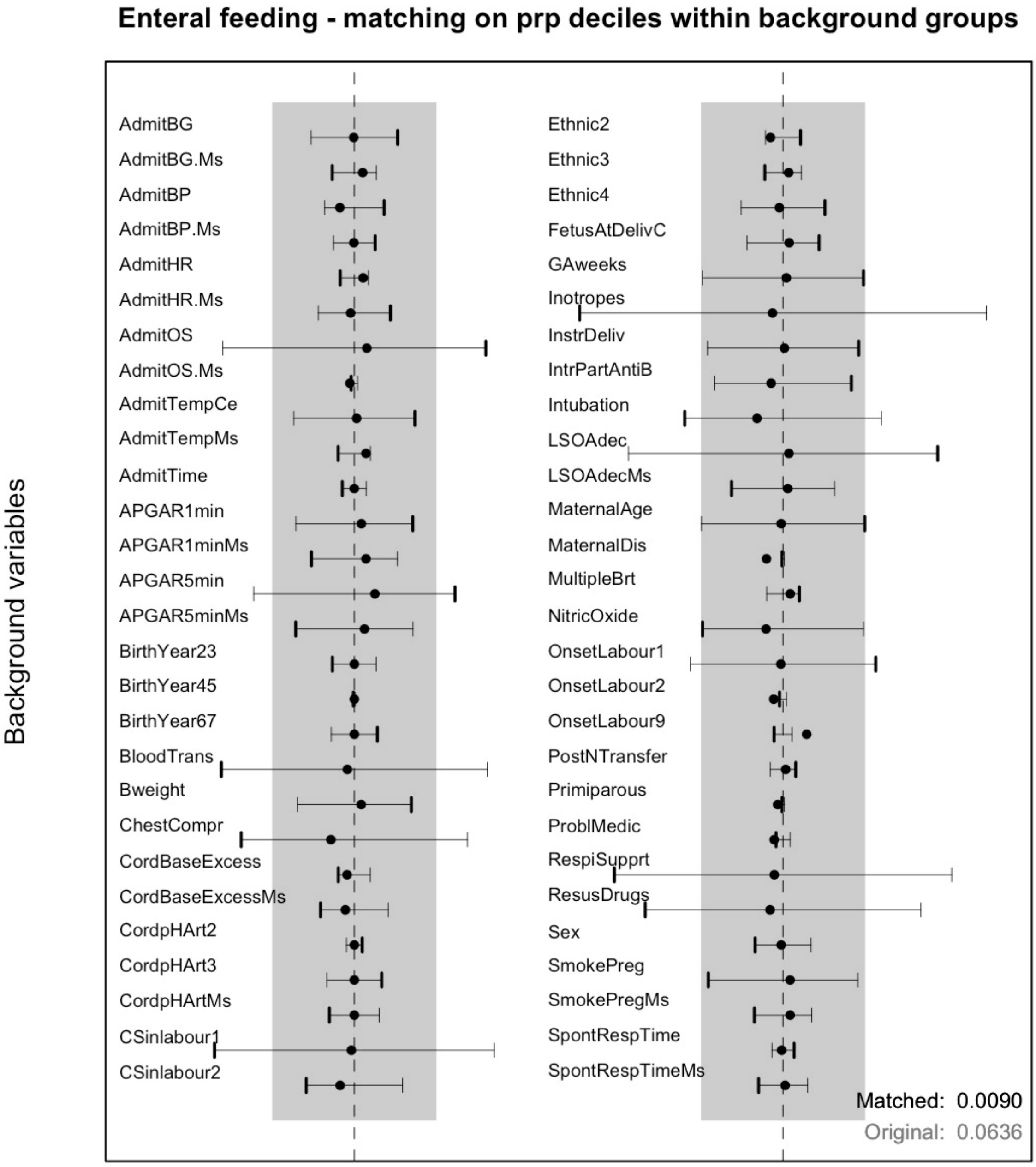

Prp=propensity; AdmitBG=admission glucose; Ms= data for this item were missing; AdmitBP=admission blood pressure;; AdmitHR=admission heart rate; AdmitOS=admission?; AdmitTempCe=admission temperature; AdmitTime=admission time; APGAR=Apgar score; min=minute; BloodTrans=blood transfusion on day 1; Bweight=birthweight; ChestCompr=chest compressions at resuscitation; CordBaseExcess=umbilical cord base excess; CordpHArt=Umbilican arterial pH; CSinLabour=In-labour Caesarean section; FetusAtDelivC=presentation of fetus at delivery; GAweeks=gestational age in weeks; InstrDeliv=Instrumental delivery; IntrPartAntiB=intrapartum antibiotics; LSOAdec=Lower Super Output Area decile; MaternalDis=maternal obstetric condition; MulipleBrt=multiple birth set; OnsetLabour=spontaneous/induced labour; PostNTransfer=postnal transfer; ProbMedic=maternal medical condition in pregnancy; RespiSupprt=Received respiratory support on day of admission; ResusDrugs=received drugs during resuscitation; SmokePreg=Maternal smoking in pregnancy; SpontRespTime=time to first breath

Supplemental table 4: Estimates of the effect of receiving enteral feeds for binary and continuous outcomes from sensitivity analyses. Results averaged over the 25 replications of the matching procedure

Variable	Sensitivity analysis		
	**Years 12-17**	**Intervention redefined**	**Inclusion of parenteral nutrition on day 1 in propensity score**
Binary outcomes: Estimate of rate difference [95% CI] (p-value)			
**NEC (pragmatic definition)**	-0·7 [-1·3, -0·1] (0·03)	-0·6 [-1·2, -0·1] (0·07)	-0·5 [-1·0, -0·0] (0·15)
**Late onset BSI (NNAP definition)**	-0·4 [-0·8, -0·003] (0·05)	-0·3 [-0·7, 0·1] (0·22)	-0·3 [-0·6, -0·0] (0·15)
**Late onset BSI (pragmatic definition)**	-11·1 [-13·7, -8·4] (<0·001)	-11·3 [-13·9, -8·7] (<0·001)	-11·4 [-13·8, -9·1] (<0·001)
**Hypoglycaemia**	-1·3 [-3·7, 1·2] (0·31)	-0·5 [-2·8, 1·9] (0·83)	-1·1 [-3·3, 1·1] (0·52)
**Survival at discharge**	4·8 [3·3, 6·3] (<0·001)	4·4 [2·9, 5·9] (<0·001)	4·5 [3·2, 5·8] (<0·001)
**Breastfeeding at discharge**	8·3 [5·0, 11·5] (<0·001)	6·5 [3·4, 9·6] (0·003)	7·9 [5·0, 10·7] (<0·001)
Continuous outcomes: mean difference [95% CI] (p-value)			
**Length of stay**	-2·3 [-3·4, -1·3] (<0·001)	-2·0 [-2·9, -1·1] (<0·001)	-2·0 [-2·8, -1·1] (<0·001)
**First day of suckling at breast**	-1·3 [-1·9, -0·7] (<0·001)	-1·2 [-1·8, -0·6] (<0·001)	-1·4 [-1·9, -0·9] (<0·001)
**First day of maternal milk**	-2·2 [-2·3, -2·0] (<0·001)	-2·1 [-2·3, -2·0] (<0·001)	-2·1 [-2·2, -2·0] (<0·001)
**Duration of PN**	-0·7 [-1·2, -0·2] (0·02)	-0·7 [-1·2, -0·2] (0·02)	-0·6 [-1·0, -0·2] (0·01)
**Duration of CV line**	-1·3 [-1·6, -1·0] (<0·001)	-1·2 [-1·4, -1·0] (<0·001)	-1·1 [-1·4, -0·9] (<0·001)
**Z score of at discharge**	0·09 [0·01, 0·17] (0·06)	0·08 [0·00, 0·16] (0·13)	0·07 [0·00, 0·15] (0·12)

Abbreviations: BSI=blood stream infection; CI=confidence interval; CV=central venous; NEC=necrotising enterocolitis; NNAP=National Neonatal Audit Programme

References

1. Imbens GW, Rubin DB. *Causal Inference for Statistics, Social, and Biomedical Science. An Introduction.* New York: Cambridge University Press; 2015

United Kingdom Neonatal Collaborative leads at contributing neonatal units

Institution	Lead
Airedale General Hospital	Dr Matthew Babirecki
Arrowe Park Hospital	Dr Anand Kamalanathan
Barnet Hospital	Dr Tim Wickham
Barnsley District General Hospital	Dr Kavi Aucharaz
Basildon Hospital	Dr Aashish Gupta
Basingstoke & North Hampshire Hospital	Dr Nicola Paul
Bassetlaw District General Hospital	Dr L M Wong
Bedford Hospital	Dr Anita Mittal
Birmingham City Hospital	Dr Lindsay Halpern
Birmingham Heartlands Hospital	Dr Pinki Surana
Birmingham Women's Hospital	Dr Matt Nash
Bradford Royal Infirmary	Dr Sunita Seal
Broomfield Hospital, Chelmsford	Dr Ahmed Hassan
Calderdale Royal Hospital	Dr Karin Schwarz
Chelsea & Westminster Hospital	Dr Shu-Ling Chuang
Chesterfield & North Derbyshire Royal Hospital	Dr Aiwyne Foo
Colchester General Hospital	Dr Jo Anderson
Conquest Hospital	Dr Graham Whincup
Countess of Chester Hospital	Dr Stephen Brearey
Croydon University Hospital	Dr John Chang
Cumberland Infirmary	Dr Yee Aung
Darent Valley Hospital	Dr Abdul Hasib
Darlington Memorial Hospital	Dr Mehdi Garbash
Derriford Hospital	Dr Alex Allwood
Diana Princess of Wales Hospital	Dr Pauline Adiotomre
Doncaster Royal Infirmary	Dr Nigel Brooke
Dorset County Hospital	Dr Abby Deketelaere
East Surrey Hospital	Dr K Abdul Khader
Epsom General Hospital	Dr Ruth Shephard
Frimley Park Hospital	Dr Sanghavi Rekha
Furness General Hospital	Dr Anas Olabi
George Eliot Hospital	Dr Mukta Jain
Gloucester Royal Hospital	Dr Jennifer Holman
Good Hope Hospital	Dr Pinki Surana
Great Western Hospital	Dr Stanley Zengeya
Guy's & St Thomas' Hospital	Dr Geraint Lee
Harrogate District Hospital	Dr Sobia Balal
Hereford County Hospital	Dr Cath Seagrave
Hillingdon Hospital	Dr Tristan Bate
Hinchingbrooke Hospital	Dr Hilary Dixon
Homerton Hospital	Dr Narendra Aladangady
Hull Royal infirmary	Dr Hassan Gaili
Ipswich Hospital	Dr Matthew James
James Cook University Hospital	Dr M Lal
James Paget Hospital	Dr Ambadkar
Kettering General Hospital	Dr Poornima Pandey
Kings College Hospital	Dr Ravindra Bhat
King's Mill Hospital	Dr Simon Rhodes
Kingston Hospital	Dr Vinay Pai
Lancashire Women and Newborn Centre	Dr Savi Sivashankar
Leeds Neonatal Service	Dr Lawrence Miall
Leicester General Hospital	Dr Jonathan Cusack
Leicester Royal Infirmary	Dr Venkatesh Kairamkonda
Leighton Hospital	Dr Michael Grosdenier
Lincoln County Hospital	Dr Ajay Reddy
Lister Hospital	Dr J Kefas
Liverpool Women's Hospital	Dr Christopher Dewhurst
Luton & Dunstable Hospital	Dr Jennifer Birch
Macclesfield District General Hospital	Dr Gail Whitehead
Manor Hospital	Dr Krishnamurthy
Medway Maritime Hospital	Dr Ghada Ramadan
Milton Keynes General Hospital	Dr I Misra
Musgrove Park Hospital	Dr Chris Knight
New Cross Hospital	Dr Rob Negrine
Newham General Hospital	Dr Imdad Ali
Nobles Hospital	Dr Prakash Thiagarajan
Norfolk & Norwich University Hospital	Dr Mark Dyke
North Devon District Hospital	Dr Michael Selter
North Manchester General Hospital	Dr P Kamath
North Middlesex University Hospital	Dr Neeraj Jain
Northampton General Hospital	Dr Subodh Gupta
Northumbria Specialist Emergency Care Hospital	Laura Winder

Northwick Park Hospital	Dr Richard Nicholl
Nottingham City Hospital	Dr Steven Wardle
Nottingham University Hospital (QMC)	Dr Steven Wardle
Ormskirk District General Hospital	Dr Andreea Bontea
Oxford University Hospitals, John Radcliffe Hospital	Dr Eleri Adams
Peterborough City Hospital	Dr Katharine McDevitt
Pilgrim Hospital	Dr Ajay Reddy
Pinderfields General Hospital	Dr David Gibson
Poole General Hospital	Prof Minesh Khashu
Princess Alexandra Hospital	Dr Chinnappa Reddy
Princess Anne Hospital	Dr Mark Johnson
Princess Royal Hospital	Dr P Amess
Princess Royal Hospital	Dr Deshpande
Princess Royal University Hospital	Dr Elizabeth Sleight
Queen Alexandra Hospital	Dr Charlotte Groves
Queen Charlotte's Hospital	Dr Lidia Tyszcuzk
Queen Elizabeth Hospital, Gateshead	Dr Anne Dale
Queen Elizabeth Hospital, King's Lynn	Dr Glynis Rewitzky
Queen Elizabeth Hospital, Woolwich	Dr Olutoyin Banjoko
Queen Elizabeth the Queen Mother Hospital	Dr Bushra Abdul-Malik
Queen's Hospital, Burton on Trent	Dr Dominic Muogbo
Queen's Hospital, Romford	Dr Khalid Mannan
Rosie Maternity Hospital, Addenbrookes	Dr Angela D'Amore
Rotherham District General Hospital	Dr Shameel Mattara
Royal Albert Edward Infirmary	Dr Christos Zipitis
Royal Berkshire Hospital	Dr Peter De Halpert
Royal Bolton Hospital	Dr Paul Settle
Royal Cornwall Hospital	Dr Paul Munyard
Royal Derby Hospital	Dr John McIntyre
Royal Devon & Exeter Hospital	Dr Chrissie Oliver
Royal Hampshire County Hospital	Dr Lucinda Winckworth
Royal Lancaster Infirmary	Dr Joanne Fedee
Royal Oldham Hospital	Dr Natasha Maddock
Royal Preston Hospital	Dr Richa Gupta
Royal Stoke University Hospital	Dr Jyoti Kapur
Royal Surrey County Hospital	Dr Ben Obi
Royal Sussex County Hospital	Dr P Amess
Royal United Hospital	Dr Stephen Jones
Royal Victoria Infirmary	Dr Naveen Athiraman
Russells Hall Hospital	Dr Chandan Gupta
Salisbury District Hospital	Dr Jim Baird
Scarborough General Hospital	Dr Kirsten Mack
Scunthorpe General Hospital	Dr Pauline Adiotomre
Southend Hospital	Dr Vineet Gupta
Southmead Hospital	Dr Alison Pike
St George's Hospital	Dr Charlotte Huddy
St Helier Hospital	Dr Ralf Hartung
St Mary's Hospital, Isle of Wight	Dr Akinsola Ogundiya
St Mary's Hospital, London	Dr Lidia Tyszcuzk
St Mary's Hospital, Manchester	Dr Ngozi Edi-Osagie
St Michael's Hospital	Dr Pamela Cairns
St Peter's Hospital	Dr Peter Martin
St Richard's Hospital	Dr Nick Brennan
Stepping Hill Hospital	Dr Carrie Heal
Stoke Mandeville Hospital	Dr Sanjay Salgia
Sunderland Royal Hospital	Dr Majd Abu-Harb
Tameside General Hospital	Dr Jacqeline Birch
The Jessop Wing, Sheffield	Dr Porus Bastani
The Royal Free Hospital	Dr Marice Theron
The Royal London Hospital	Dr Vadivelam Murthy
Torbay Hospital	Dr Siba Paul
Tunbridge Wells Hospital	Dr Hamudi Kisat
University College Hospital	Dr Giles Kendall
University Hospital Coventry	Dr Puneet Nath
University Hospital Lewisham	Dr Ozioma Obi
University Hospital of North Durham	Dr Mehdi Garbash
University Hospital of North Tees	Dr Hari Kumar
Victoria Hospital, Blackpool	Dr Chris Rawlingson
Warrington Hospital	Dr Delyth Webb
Warwick Hospital	Dr Bird
Watford General Hospital	Dr Sankara Narayanan
West Cumberland Hospital	Dr Yee Mon Aung
West Middlesex University Hospital	Dr Eleanor Hulse
West Suffolk Hospital	Dr Ian Evans
Wexham Park Hospital	Dr Rekha Sanghavi

Whipps Cross University Hospital	Dr Caroline Sullivan
Whiston Hospital	Dr Ros Garr
Whittington Hospital	Dr Wynne Leith
William Harvey Hospital	Dr Vimal Vasu
Worcestershire Royal Hospital	Dr Liza Harry
Worthing Hospital	Dr Katia Vamvakiti
Wythenshawe Hospital	Dr Ngozi Edi-Osagie
Yeovil District Hospital	Dr Megan Eaton
York District Hospital	Dr Sundeep Sandhu
Aberdeen Maternity Hospital,	Dr Saulius Satas
Borders General Hospital, Melrose	Dr Clare Irving
Dumfries and Galloway Royal Infirmary	Dr Andrew Eccleston
Cross House Hospital, Kilmarnock	Dr S Kinmond
Dr Gray's Hospital, Elgin	Dr Shelagh Parkinson
Forth Valley Hospital, Larbert	Dr Dominic O'Reilly
Ninewells Hospital, Dundee	Dr Bhushan
Princess Royal Maternity Hospital, Glasgow	Dr Andrew Powls
Raigmore Hospital, Inverness	Dr P Van Der Heide
Royal Alexandra Hospital, Paisley	Dr Hilary Conetta
The Queen Elizabeth University Hospital Glasgow	Dr J Coutts and Dr AM Heuchan
Simpsons Centre for Reproductive Health, Royal Infirmary of Edinburgh	Professor Ben Stenson
St John's Hospital, Livingston	Helen Rhodes
Victoria Hospital, Kirkcaldy	Dr Sean Ainsworth
Wishaw General Hospital	Dr Caroline Delahunty
Aberdeen Maternity Hospital,	Dr Saulius Satas
Singleton Hospital	
Princess of Wales Hospital	Dr Arun Ramachandran
Royal Gwent Hospital	Dr Kate Creese
Nevill Hall Hospital	Dr Sunil Reddy
Glan Clwyd Hospital	Dr Sunil Reddy
Wrexham Maelor Hospital	Dr Ian Barnard
Ysbyty Gwynedd	Dr Brendan Harrington
University Hospital of Wales	Dr Mike Cronin
Prince Charles Hospital	Dr Nitin Goel
Glangwili General Hospital	Dr Iyad Al-Muzaffar
Withybush Hospital	Dr Prem Pitchaikani
